# Alumni Association of Buffalo

**Published:** 1871-11

**Authors:** 


### Alumni Association of Buffalo.

This Association was formed in February, 1871, with the object of obtaining, and keeping in some suitable form, the addresses and all matters of historic interest concerning the graduates of the Buffalo Medical College. Dr. T. D. Strong, of Westfield, N.Y., was elected President, and Dr. W. W. Miner, Secretary.

In order to fully accomplish the object of the Association, all graduates of the University of Buffalo Medical Department will confer a favor by sending their address and any matters of interest in their histories to the Secretary of the Alumni Association, Dr. W. W. Miner, Office of the Buffalo Medical and Surgical Journal, Buffalo, N. Y.

